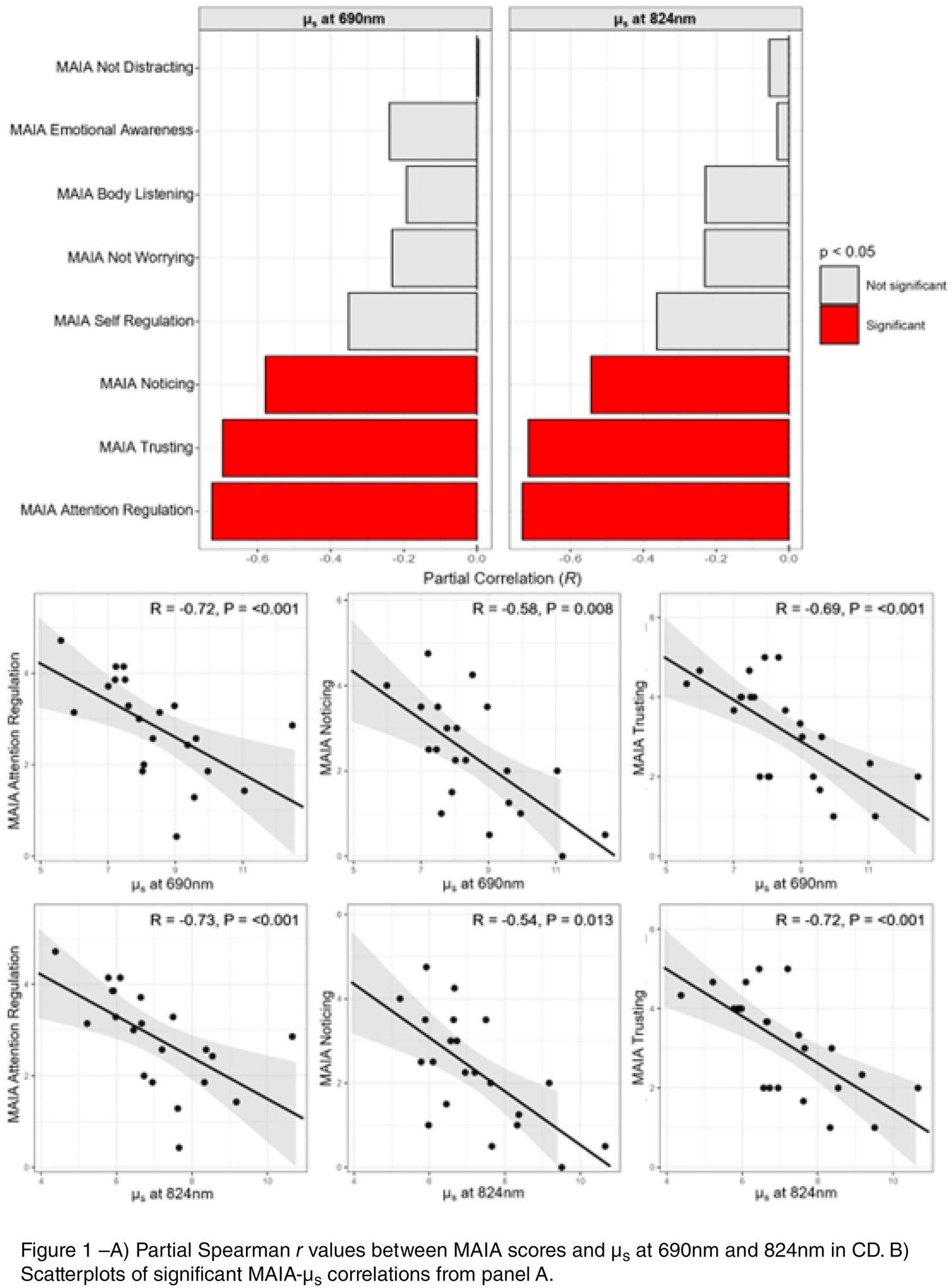# Poster Session II - A226 BRAIN NEAR INFRARED SPECTROSCOPY REVEALS A POTENTIAL LINK BETWEEN PREFRONTAL CORTEX HYPOXIA, MITOCHONDRIAL DYSFUNCTION, AND ALTERED INTEROCEPTION IN CROHN’S DISEASE

**DOI:** 10.1093/jcag/gwaf042.226

**Published:** 2026-02-13

**Authors:** A Hansen, A Soroush, C Ma, C Diribe, A Fuhrmann, D Marshall, C Lu, C Seow, R Ingram, K Novak, G G Kaplan, R Panaccione, J F Dunn, M G Swain

**Affiliations:** University of Calgary Cumming School of Medicine, Calgary, AB, Canada; University of Calgary Cumming School of Medicine, Calgary, AB, Canada; University of Calgary Cumming School of Medicine, Calgary, AB, Canada; University of Calgary Cumming School of Medicine, Calgary, AB, Canada; University of Calgary Cumming School of Medicine, Calgary, AB, Canada; University of Calgary Cumming School of Medicine, Calgary, AB, Canada; University of Calgary Cumming School of Medicine, Calgary, AB, Canada; University of Calgary Cumming School of Medicine, Calgary, AB, Canada; University of Calgary Cumming School of Medicine, Calgary, AB, Canada; University of Calgary Cumming School of Medicine, Calgary, AB, Canada; University of Calgary Cumming School of Medicine, Calgary, AB, Canada; University of Calgary Cumming School of Medicine, Calgary, AB, Canada; University of Calgary Cumming School of Medicine, Calgary, AB, Canada; University of Calgary Cumming School of Medicine, Calgary, AB, Canada

## Abstract

**Background:**

Crohn’s disease (CD) patients show heightened interoceptive awareness (perception of internal body sensations), which may worsen psychological distress. The prefrontal cortex (PFC) plays a key role in processing interoceptive signals. PFC hypoxia can be measured as low tissue oxygen saturation (S_t_O_2_) by frequency-domain near-infrared spectroscopy (FD-NIRS). Mitochondria are key cellular oxygen consumers, and hypoxia can alter their function/structure. NIRS light scattering (NIRS-LS) can detect mitochondrial dysfunction *in vivo*. Thus, we hypothesized that low PFC oxygenation could lead to mitochondrial dysfunction and altered interoception in CD.

**Aims:**

To investigate whether PFC NIRS-LS is altered in CD patients compared to healthy controls (HCs) and examine its relationship to PFC hypoxia and interoceptive awareness.

**Methods:**

HCs (*n*=15, age 44±15) and CD patients (*n*=26, age 49±14) were recruited. CD patients were grouped as remitted (rCD; *n*=16, age 51±14) and active disease (aCD; *n*=10, age 46±15) using the Harvey-Bradshaw Index. Resting PFC FD-NIRS was measured to obtain S_t_O_2_ and NIRS-LS (measured as µ_s_ at 690nm and 824nm; wavelengths sensitive to the absorption spectra of oxyhemoglobin and deoxyhemoglobin). Interoceptive awareness was assessed in the CD cohort using the Multidimensional Assessment of Interoceptive Awareness (MAIA) questionnaire. µ_s_ was compared between HC, rCD and aCD (ANCOVA, age-controlled) and correlated with PFC S_t_O_2_ (partial Pearson, HC & CD) and MAIA scores (partial Spearman, CD), controlling for disease activity (aCD vs rCD).

**Results:**

µ_s_ at 690nm or 824nm did not differ significantly between HC, rCD and aCD (Table 1). In the overall CD cohort, there was a moderate positive correlation between S_t_O_2_ and µ_s_ at both wavelengths (*r*=0.4, *p*=0.03 for both). This relationship was not seen in HC. In CD, µ_s_ at 690nm and 824nm correlated moderately/strongly and negatively with MAIA subscales: *noticing* (*r*=-0.6 and -0.5, respectively, *p*=0.01 for both), *attention regulation* (*r*=-0.7, *p*<0.001 for both), and *trusting* (*r*=-0.7, *p*<0.001 for both) (Fig. 1).

**Conclusions:**

Reduced PFC NIRS-LS correlated with lower PFC oxygenation and higher MAIA scores in CD patients. Lower µ_s_ occurs in ischemic brain injury and is reported to reflect mitochondrial dysfunction. Our findings suggest a potential link between PFC hypoxia, mitochondrial dysfunction, and enhanced interoception in CD.

**Funding Agencies:**

CIHR